# rAAV expressing recombinant neutralizing antibody for the botulinum neurotoxin type A prophylaxis

**DOI:** 10.3389/fmicb.2022.960937

**Published:** 2022-09-27

**Authors:** Artem A. Derkaev, Ekaterina I. Ryabova, Ilias B. Esmagambetov, Dmitry V. Shcheblyakov, Svetlana A. Godakova, Irina D. Vinogradova, Anatoly N. Noskov, Denis Y. Logunov, Boris S. Naroditsky, Alexander L. Gintsburg

**Affiliations:** Federal State Budget Institution “National Research Center for Epidemiology and Microbiology Named After Honorary Academician N.F. Gamaleya” of the Ministry of Health of the Russian Federation, Moscow, Russia

**Keywords:** adeno-associated viral vector (AAV), single-domain antibodies, VHH, botulinum neurotoxin, *Clostridium botulinum*, botulism, viral vector

## Abstract

Botulinum neurotoxin (BoNT) is one of the most dangerous bacterial toxins and a potential biological weapon component. BoNT mechanism of pathological action is based on inhibiting the release of neurotransmitters from nerve endings. To date, anti-BoNT therapy is reduced to the use of horse hyperimmune serum, which causes many side effects, as well as FDA-approved drug BabyBig which consists of human-derived anti-BoNT antibodies (IgG) for infant botulinum treatment. Therapeutics for botulism treatment based on safer monoclonal antibodies are undergoing clinical trials. In addition, agents have been developed for the specific prevention of botulism, but their effectiveness has not been proved. In this work, we have obtained a recombinant adeno-associated virus (rAAV-B11-Fc) expressing a single-domain antibody fused to the human IgG Fc-fragment (B11-Fc) and specific to botulinum toxin type A (BoNT/A). We have demonstrated that B11-Fc antibody, expressed *via* rAAV-B11-Fc treatment, can protect animals from lethal doses of botulinum toxin type A, starting from day 3 and at least 120 days after administration. Thus, our results showed that rAAV-B11-Fc can provide long-term expression of B11-Fc-neutralizing antibody *in vivo* and provide long-term protection against BoNT/A intoxication. Consequently, our study demonstrates the applicability of rAAV expressing protective antibodies for the prevention of intoxication caused by botulinum toxins.

## Introduction

Botulinum neurotoxin, produced by the spore-forming bacterium *Clostridium botulinum*, is one of the strongest organic poisons known. Currently, seven serotypes of botulinum neurotoxin are characterized, of which A, B, and E are the most toxic. Toxins most often enter the human body with low-quality canned food consumption. Mortality can reach up to 5%−10% in developing countries (Godakova et al., 2019). Immunity is not usually formed against foodborne botulism; there are known cases of people suffering from botulism several times in a lifetime.

Equine anti-botulinum serum is the only specific therapy for botulism, being very effective in rapid diagnosis and early treatment. However, equine hyperimmune serum can cause serum sickness and other hyperimmune reactions in the body (Vanella de Cuetos et al., 2011).

The existing pentavalent vaccine against botulinum toxin serotypes A, B, C, D, and E is not currently used due to unproven efficacy and side effects (Godakova et al., 2019). Thus, there are no licensed products for specific botulism prophylaxis at present. Therefore, prevention agents against botulism need to be developed.

Antibodies can specifically bind the corresponding antigens with high selectivity. As research results show, immunoglobulins are most effective in the treatment of viral pathology and bacterial infections accompanied by the production of exotoxins, including tetanus, diphtheria, and botulism (Lucchi et al., 2021). There are several examples of human monoclonal antibody-based therapeutics against some botulinum toxin serotypes, which have demonstrated safety and efficacy and are currently at various stages of clinical research (Nowakowski et al., 2002; Fan et al., 2017; Rasetti-Escargueil et al., 2017; Garcia-Rodriguez et al., 2018; Snow et al., 2019; Matsumura et al., 2020). Along with monoclonal antibodies, studies are using single-domain antibodies (VHH) (Esmagambetov et al., 2021; Ryabova et al., 2021). Such antibodies can bind hard-to-reach antigenic epitopes through long parts of the variable chains - CDR3s. VHH fusion with the canonical immunoglobulin Fc fragment allows VHH-Fc to significantly increase their neutralizing activity and serum half-life (Godakova et al., 2019). It has been shown in particular that HC-only antibodies or VHHs derived from HC-only antibodies produced by camelids demonstrate protection not only against various non-toxin diseases (Shcheblyakov et al., 2019; Favorskaya et al., 2022) but also strong anti-BoNT activities in animal models (Yu et al., 2009; Swain et al., 2010; Tremblay et al., 2010) comparable to those of canonical antibodies (Garcia-Rodriguez et al., 2011).

Nevertheless, antibodies circulate in the organism for a limited period (Godakova et al., 2019). Thus, it is necessary to develop a biological agent that enables the long-term circulation of antibodies in the organism to provide long-term protection in prevention mode. Transfer of genes of therapeutic antibodies *via* viral vector transduction represents an attractive strategy for long-term protection in this case. Development of viral vectors with improved safety characteristics and greater efficiency of transgene delivery into cells, as well as provision of long-term and tissue-specific expression of protective antibodies, maybe promising.

Antibody molecules can be expressed through a variety of viral vectors, including baculovirus, rhabdovirus, vaccinia virus, adenovirus, and adeno-associated virus (Nieto and Salvetti, 2014). In this article, we focused on antibody delivery using a recombinant adeno-associated virus (rAAV) (Bulcha et al., 2021). rAAV are the most promising delivery vehicles due to their non-pathogenic properties, lack of immunogenicity, and inability to integrate into the genome, as well as long-term persistence in the body (Nieto and Salvetti, 2014).

In our previous study, we obtained a single-domain antibody fused to a human Fc fragment (B11-Fc) with neutralizing activity against botulinum toxin type A (BoNT/A) (Godakova et al., 2019). In this study, we used a recombinant adeno-associated virus, containing the gene of the B11-Fc antibody (rAAV-B11-Fc), to provide a long-term expression of B11-Fc antibody *in vivo*, and as result to induce a long-term protection against a lethal dose of BoNT/A.

## Materials and methods

### Animal housing conditions

Female BALB/c mice (6 weeks old, weighing 18–20 g) were purchased from “Pushchino breeding facility” (Pushchino, Moscow, Russia) accredited by the Association for Assessment and Accreditation of Laboratory Animal Care (AAALAC International) and maintained at the central animal facility of the Gamaleya Research Center for Epidemiology and Microbiology. Mice were kept at constant temperature (22 ± 2°C) and relative humidity (50%) with 12 h of artificial light per day, housed in individual T2-type cages (eight animals per cage), and fed with dried food and water *ad libitum*. Mice were observed every 2 h post-injection except night time for a week. Animals showing typical symptoms of botulism, including muscle paralysis and respiratory difficulty, were euthanized by cervical dislocation.

### Plasmid design

The AAV-DJ Packaging System (Cell Biolabs, USA) with the pAAV-DJ Vector, pHelper Vector, and pAAV-GFP Control Vector plasmids were used to construct plasmids for rAAV-B11-Fc production. The pAAV-DJ Vector contains the *rep* genes required for replication and the *cap* genes encoding capsid proteins. The pHelper Vector contains most of the adenoviral genome required for infectious rAAVs assembly (i.e., genes *E2A, E4*, and *VA* RNA).

The technique of Fc-fused single-domain antibody development was described previously (Godakova et al., 2019; Esmagambetov et al., 2021; Voronina et al., 2021; Favorskaya et al., 2022). The nucleotide sequence encoding B11-Fc antibody was synthesized at Evrogen Company (Moscow, Russia) and cloned into the pAAV–EGFP Control Vector plasmid instead of the *EGFP* gene at the EcoRI and XbaI restriction sites, thus obtaining the pAAV-B11-Fc plasmid.

### Production of an rAAV–B11-Fc expressing B11-Fc antibody

The process of rAAV production was described previously (Ryabova et al., 2021). Briefly, Plasmid Select Xtra Starter Kit (Cytiva Life Sciences, USA) was used to obtain the supercoiled form of plasmid DNA. The quantitative measurement of DNA was carried out on a NanoDrop 2000C spectrophotometer (Thermo Fisher Scientific, USA) at a wavelength of 260 nm. Restriction hydrolysis and further electrophoresis in 1% agarose gel were performed to verify the authenticity of the plasmid DNA for comparison with the theoretical length of the restricted fragments.

rAAV–B11-Fc was produced by transient transfection of HEK 293 cells (obtained from the cell cultures collection of the Gamaleya National Research Center for Epidemiology and Microbiology) and cultured in a BioFlo 320 bioreactor (Eppendorf, Germany) with a BioBLU 5c disposable vessel (Eppendorf, Germany) filled with Fibra-Cel matrix (Eppendorf, Germany). pAAV–DJ Vector, pAAV–DJ-Helper, and pAAV–B11-Fc were used in a 1:1:1 ratio for transfection. Transfection was performed with a polyethyleneimine (PEI) transfecting agent in a ratio of 1:4 [DNA (μg) : PEI (μl)]. After 72 h of cultivation, the cell suspension was processed through the addition of polysorbate 20 (1%), 2 mM MgCl_2_, and 20 U/ml of Benzonase (Merck Millipore, USA) for 4 h.

rAAV–B11-Fc purification was performed using an AKTA flux S tangential flow filtration system (Cytiva Life Sciences, USA) and a Hollow Fiber Cartridge, 100 kDa (Cytiva Life Sciences, USA) following affinity chromatography using AVB Sepharose resin (Cytiva Life Sciences, USA) according to the manufacturer's protocol.

### Evaluation of rAAV–B11-Fc characteristics

The purity of the obtained rAAV–B11-Fc was assessed by SDS-PAGE with 4%−20% Mini-PROTEAN TG Precast Protein Gel (Bio-Rad, USA) under reducing conditions.

The identity of the rAAV–B11-Fc was assessed by immunoblotting. The samples were separated by SDS-PAGE with 4%−20% Mini-PROTEAN TG Precast Protein Gel (Bio-Rad, USA) under reducing conditions. Protein transfer to the membrane (Amersham Protran Premium 0.45 μm NC, GE Healthcare Life science, USA) was performed using the Trans-Blot Turbo Transfer System. The membrane was blocked with 5% skimmed milk solution (Sigma–Aldrich, USA) in PBS-T. Primary antibodies – Adeno-Associated Virus 2/AAV2 (VP1 + VP2 + VP3) Rabbit Polyclonal Antibody (OriGene, USA) – were added in a ratio of 1:1,000 and the membrane was rinsed three times in PBS-T. Afterward HRP-conjugated Anti-Rabbit IgG (Sigma–Aldrich, USA) was added in a ratio of 1:2,500, rinsed five times in PBS-T, and visualized using ECL Substrate (Bio-Rad, USA) and Amersham Imager 600 (GE Healthcare, USA).

The number of rAAV–B11-Fc genomic copies was determined by the AAVpro Titration Kit (for Real Time PCR) Ver.2 according to the manufacturer's protocol.

HEK293 cells were treated with purified rAAV–B11-Fc to evaluate the transducing ability and expression of B11-Fc transgene. Cells were seeded on a 96-well plate in a DMEM medium (4 mM glutamine, 10% FBS, sodium bicarbonate 3.8 g/L) in a 100 μl volume with a concentration of 0.5 × 10^6^ viable cells/ml. After 4 h, 10 μl of purified rAAV–B11-Fc per well, were added to the plate. After 48 h, the culture medium was taken for further analysis. The culture medium was evaluated for the presence of B11-Fc antibody *via* immunoblotting, using Anti-Human IgG (Fc specific) Peroxidase-conjugated antibody (A0170-1ML, Sigma–Aldrich, USA) at a ratio of 1:2,500.

### Botulinum toxin A preparation

**Figure 1:**
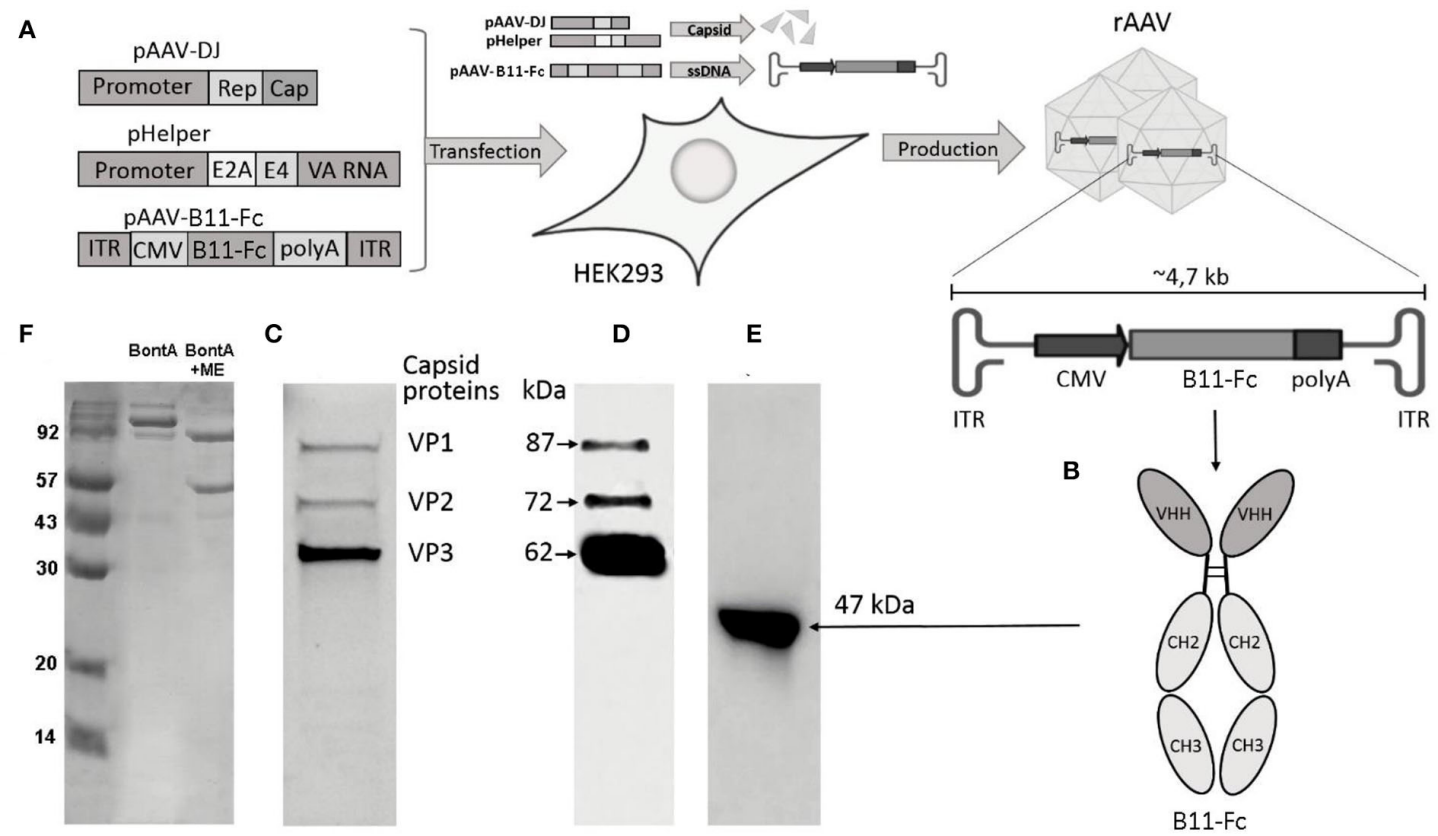
Key methods and experimental results. **(A)** Scheme of rAAV-B11-Fc production. Plasmids pAAV-DJ, pHelper, and pAAV-B11-Fc are cotransfected in HEK293 cells for rAAV-B11-Fc production; **(B)** Diagram of B11-Fc antibody structure; **(C)** SDS–PAGE of purified rAAV-B11-Fc under reducing conditions; **(D)** Immunoblot of purified rAAV-B11-Fc under reducing conditions with specific antibodies to the capsid proteins anti-VP1, VP2, and VP3; **(E)** Culture medium from HEK293 cells transduced with purified rAAV-B11-Fc; **(F)** SDS–PAGE of BoNT/A at reducing and non-reducing conditions.

Botulinum toxin A was obtained using *C. botulinum* strain A98. The strain was cultivated under anaerobic conditions for 5 days. The bacterial suspension was precipitated by centrifugation at 5,000 × g for 30 min at 10°C. Bacterial culture proteins from the filtrate were concentrated by acid precipitation at pH = 3.8 for 45 min. The precipitate was separated by centrifugation at 12,000 × g for 30 min at 10°C and dissolved in 47 mM citrate–phosphate buffer with pH = 5.6. S300 gel filtration and ion-exchange chromatography were performed on the AKTA start system (GE Healthcare Lifesciences, USA) with DE cellulose (Pharmacia, Sweden). The toxin (90–95%, 150 kDa) was additionally purified by chromatography with DE cellulose (Pharmacia, Sweden) in borate buffer with pH = 8 and eluted with 50 mM NaCl. The specific antigenic activity of BoNT/A was determined by reaction with monospecific antibodies to BoNT/A (Gamaleya Laboratory of Clostridiosis and commercial preparation of the Scientific Center for Expert Evaluation of Medicinal Products Russian Federation). Purified BoNT/A was checked by SDS-electrophoresis with 2-mercaptoethanol (Figure 1F). The toxin was filtered through 0.22-μm syringe filter before injection.

The BoNT/A concentration was determined spectrophotometrically (UV 280 nm) and calorimetrically (Bradford protein analysis). LD50 was determined on 6-week-old female BALB/c mice (weighing 18–20 g). Specific activity was determined by the standard mouse lethality assay method described by Miia Lindström and Hannu Korkeala (Lindström and Korkeala, 2006). The specific activity ranged from 10 to 30 pg/mouse among lots of BoNT/A. Variations in the specific activity of BoNT/A were associated with different batches of animals and BoNT/A. Therefore, we checked the specific toxic activity before each experiment and for each batch of animals and toxin.

### Evaluation of toxic signs

The severity of toxic signs was assessed according to the standard method (Vazquez-Cintron et al., 2020) by three investigators who were unaware of the treatment groups. The following rating scale was used to clinically assess botulinum indication: mild abdominal paradox (one point), moderate abdominal paradox (two points), or severe abdominal paradox and/or agonal breathing (three points); salivation and lethargy (1 point), weakness (two points), or complete paralysis of the body (no recovery reflex, three points). Animals that scored 12 points during consecutive observations were euthanized and the deceased were given 16 points.

### Evaluation of rAAV–B11-Fc protective capacities *in vivo*

A total of 80 BALB/c female mice weighing 18–20 g were treated with a single intramuscular injection of rAAV–B11-Fc with concentrations of 10^8^, 10^9^, 10^10^, and 10^11^ gc/mouse, with 20 mice per group. Thereafter, mice received 10 LD50 of BoNT/A (200 μl) intraperitoneally, on days 3, 7, 10, 14, and 21 after administration of each dose of rAAV–B11-Fc, four mice in each subgroup. Mice were observed one time a day for 1 week. A specific pathological pattern (dystonia's abdominal muscles, waistline decreasing) was observed in sick mice. The positive control groups of 10 mice received the rabbit antitoxin IgG, and the negative control groups of 10 mice received a standard saline solution.

To study the effectiveness of the rAAV–B11-Fc selected dose, four groups of four mice were used. Animals were challenged with 10 LD50 of BoNT/A and treated with rAAV–B11-Fc 24, 48, and 72 h before the challenge (first three groups, respectively). The fourth group of untreated animals challenged with 10 LD50 was used as a control group. Mice were observed for symptoms of botulism within 80 h of intoxication.

To estimate the duration of the protection for rAAV–B11-Fc selected doses, 13 experimental groups and 13 control groups of animals (four BALB/c mice per group) were used. All mice in 13 experimental groups were treated with 10^11^ gc/animal of rAAV–B11-Fc and all animals in 13 control groups were untreated. Then, experimental and control groups were assigned to the intraperitoneal challenge with 10 LD50 of BoNT/A on day 0, 1, 2, 3, 7, 14, 21, 45, 60, 90, 120, or 150 (**Figure 4B**).

To determine the breakthrough dose of BoNT/A exposure for rAAV–B11-Fc–treated mice, 20, 50, or 100 LD50 of BoNT/A were injected 10 days after the administration of rAAV–B11-Fc. Eight groups of mice were used for this, four animals per group each. Four groups of mice were challenged intraperitoneally with BoNT/A doses of 10, 20, 50, and 100 LD50, respectively, and four groups were challenged intravenously with BoNT/A at doses of 10, 20, 50, and 100 LD50, respectively. rAAV–B11-Fc was administrated intramuscularly at a dose of 10^11^ gc/mouse.

### Evaluation of pharmacokinetic of B11-Fc antibody expressed *via in vivo* rAAV–B11-Fc transduction

A total of 48 BALB/c female mice weighing 18–20 g were treated with a single intramuscular injection of rAAV–B11-Fc with a dose of 10^11^ gc/mouse, with four animals per group.

The blood was collected from the facial vein before rAAV–B11-Fc transduction (point 0) and 1, 2, 3, 7, 14, 21, 45, 60, 90, 120, and 150 days after rAAV–B11-Fc transduction. Serum was collected and stored at−20°C until further analysis. Serum samples were taken from four animals per time point.

The concentration of B11-Fc antibody in collected serum samples was measured by ELISA kit IgG total-EIA-BEST (Vector-Best, Russia); 10 μl/well of serum was added. The analysis was performed according to the manufacturer's instructions except second for antibodies, which were changed to ECL Human IgG, HRP-linked whole Ab (Cytiva, NA933-1ML), and antibody standards, which were changed to purified B11-Fc antibody.

### Evaluation of immunogenicity of rAAV–B11-Fc vector

Immunogenicity of rAAV–B11-Fc vector was evaluated by the detection of anti-rAAV capsid protein antibodies in the serum of rAAV–B11-Fc-treated mice. Previously collected serum samples (Section “Evaluation of pharmacokinetic of B11-Fc antibody expressed *via in vivo* rAAV-B11-Fc transduction”) were used for this analysis. To detect anti-rAAV capsid protein antibodies, purified rAAV–B11-Fc was coated on microplates (Nunc, Denmark) at 100 ng/well in 50 mM bicarbonate buffer and incubated overnight at 4°C. After rinsing three times with TPBS plates were blocked with 5% dry milk in PBS for 1 h at 37°C. Then various dilutions of serum samples were added to the wells and the plates were incubated for 1 h at 37°C. Anti-Mouse IgG (whole molecule)–Peroxidase antibodies (1:5,000; A9044, Sigma–Aldrich, USA) were used as a conjugate.

The titers of anti-rAAV capsid protein antibodies were defined as the highest dilutions of serum samples with OD450 nm values at least two times greater than OD450 nm values of control serum samples from intact mice.

### Evaluation of immunogenicity of B11-Fc antibody, expressed *via* rAAV–B11-Fc transduction

Immunogenicity of B11-Fc antibody, expressed *via* rAAV–B11-Fc transduction, was evaluated by the detection of anti-B11-Fc antibodies in the serum of rAAV–B11-Fc-treated mice. Previously collected serum samples (Section “Evaluation of pharmacokinetic of B11-Fc antibody expressed *via in vivo* rAAV-B11-Fc transduction”) were used for this analysis. To detect anti-B11-Fc antibodies, purified B11-Fc antibodies were coated on microplates (Nunc, Denmark) at 100 ng/well in 50 mM bicarbonate buffer and incubated overnight at 4°C. Further manipulations were carried out similar to those described in Section “Evaluation of immunogenicity of rAAV-B11-Fc vector”.

The titers of anti-B11-Fc antibodies were defined as the highest dilutions of serum samples with OD450nm values at least two times greater than OD450 nm values of control serum samples from intact mice.

### Statistical analysis

Data were analyzed using EXCEL 2010, Graphpad Prism 9.0, and ELISA Master (AlkorBio, Russia) software. The Mann–Whitney *U*-test and the Gehan–Wilcoxon test with a significance level of 0.05 were used to assess intergroup differences in antibody titers and animal survival. Median survival was determined using Kaplan–Meier analysis. The *p*-value was determined using the *t*-test and log-rank test.

## Results

### Production and characterization of rAAV–B11-Fc

Transient transfection of HEK293 cells with supercoiled forms of plasmids in an adhesive bioreactor BioBlu 5c was performed to produce rAAV–B11-Fc, as well as purification of rAAV–B11-Fc was performed as described previously (Ryabova et al., 2021).

The purified rAAV–B11-Fc was characterized using SDS-PAGE under reducing conditions (Figure 1C). The electropherogram showed the structural proteins of the capsid - VP1, VP2, and VP3 (1:1:10), which corresponded to the molecular weight of wild-type AAV proteins. No other host cell proteins in significant quantities were detected in rAAV–B11-Fc samples.

Next, the rAAV–B11-Fc was analyzed by immunoblotting using specific antibodies to the capsid proteins anti-VP1, VP2, and VP3 (Figure 1D), to confirm specificity. The capsid proteins VP1, VP2, and VP3 of the rAAV–B11-Fc were shown to specifically react with anti-antibodies, which confirmed their authenticity.

Quantitative PCR analysis of the rAAV–B11-Fc sample showed 8.24 × 10^12^ genomic copies/ml.

The transduction capacity of purified rAAV–B11-Fc and expression of B11-Fc antibody in the rAAV–B11-Fc transduced cells were determined *via* transduction of HEK293 cells with rAAV–B11-Fc (10 μl of sample to 100 μl of HEK293 cells in a 96-well plate). Transduction showed expression of the target B11-Fc antibody, which was confirmed by immunoblotting with specific ECL–HRP Human IgG antibodies (Figure 1E).

Thus, the obtained rAAV–B11-Fc was characterized to be of high purity, authenticity, and transducing capacity.

### Evaluation of optimal protective dose of rAAV–B11-Fc against BoNT/A *in vivo*

**Figure 2:**
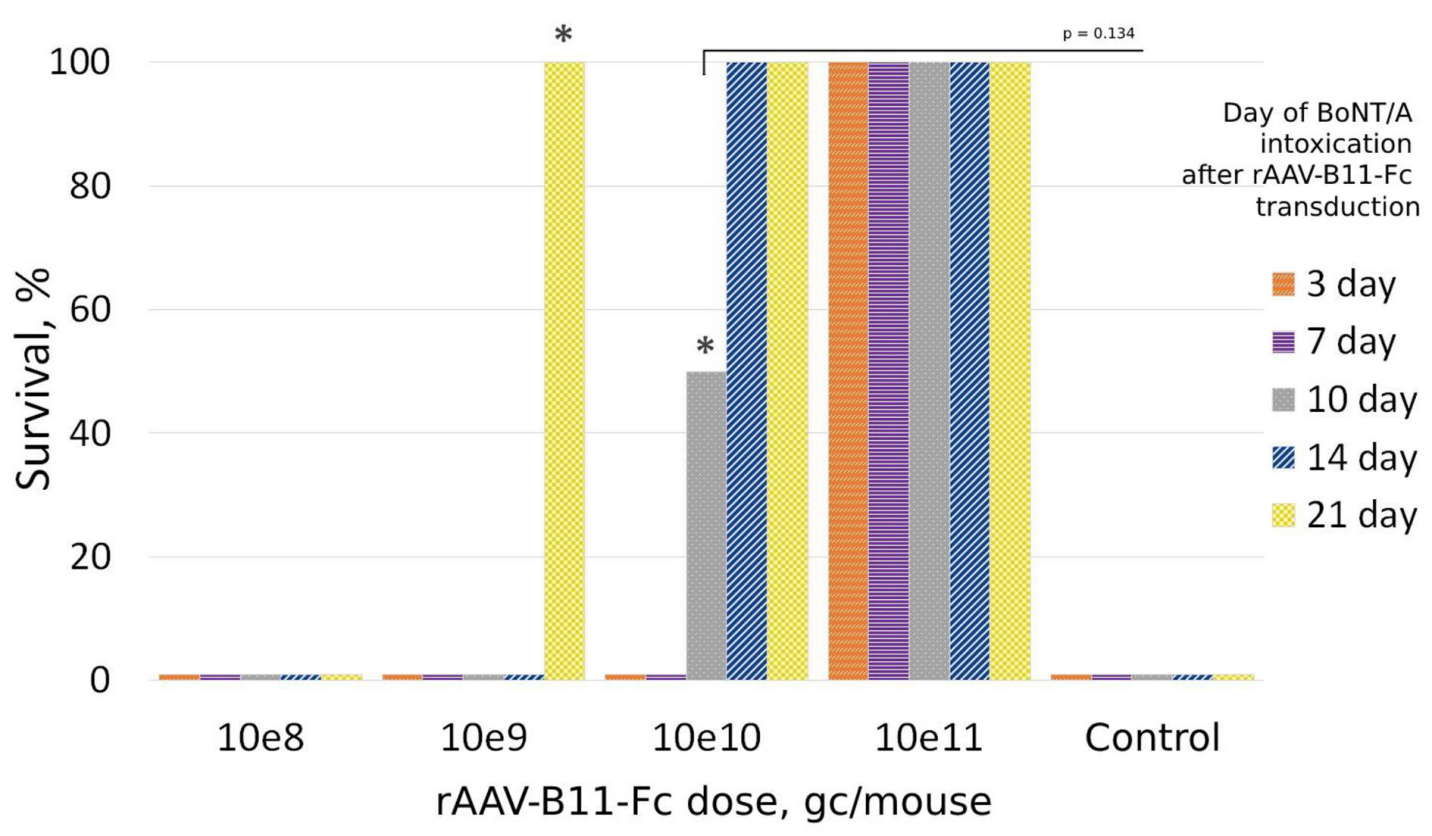
Protection of mice treated with rAAV-B11-Fc at different periods before intoxication with 10LD50 BoNT/A. Groups of 20 mice received various doses of the rAAV-B11-Fc followed by a challenge with BoNT/A at different time intervals. The bars show the percentage of survival rate. The asterisks indicate the presence of botulism symptoms in the groups.

To evaluate rAAV–B11-Fc protective capacities, the optimal protective dose of rAAV–B11-Fc was determined *in vivo*. For this purpose, mice were treated with various doses of rAAV-B11-Fc intramuscularly, and then 10 LD50 of BoNT/A was injected intraperitoneally at different time intervals. As shown in Figure 2, mice treated with 10^8^ gc/mouse dose were not protected from BoNT/A intoxication. The 10^9^ gc per animal dose was able to protect the mice from day 21, but one animal demonstrated severe symptoms of botulism (abdominal breathing). Administration of rAAV–B11-Fc at a dose of 10^10^ gc/mouse provided complete protection within 14–21 days after the injection with two mice demonstrated mild symptoms of botulism at 14 days, and weak protection at 10 days with symptoms of botulism in a survived mouse. A dose of 10^11^ gc/mouse demonstrated complete protection from day 3 after rAAV–B11-Fc injection with no visible symptoms of botulism. All untreated control mice died within 24 h of intoxication.

The survival times for the control and 10^8^ gc/mouse groups were 8 and 9.5 h, respectively. For the 10^9^ gc/mouse group: 8 h for the 3–7-day groups, 11 h for the 14-day group, and 18 h for the 21-day group. For the 10^10^ gc/mouse group: 10 h for the 3-day group.

The protective capacities of various doses of rAAV–B11-Fc were shown in this way. The most promising results were obtained with the dose of 10^11^ gc per mouse, which was able to protect the animals starting from day 3 after rAAV–B11-Fc transduction. Thus, the dose of 10^11^ gc/mouse (5^*^10^12^ gc/kg) was chosen for further experiments.

### Study of rAAV-B11-Fc protective capacities in the prevention mode

To study rAAV–B11-Fc protective properties in the prevention mode of BoNT/A intoxication, at least two *in vivo* experiments were performed. In one of them, the minimal protective time lag between rAAV–B11-Fc administration and BoNT/A intoxication was estimated, and in the other one, the duration of protection against BoNT/A, caused by a single rAAV–B11-Fc injection was determined.

**Figure 3:**
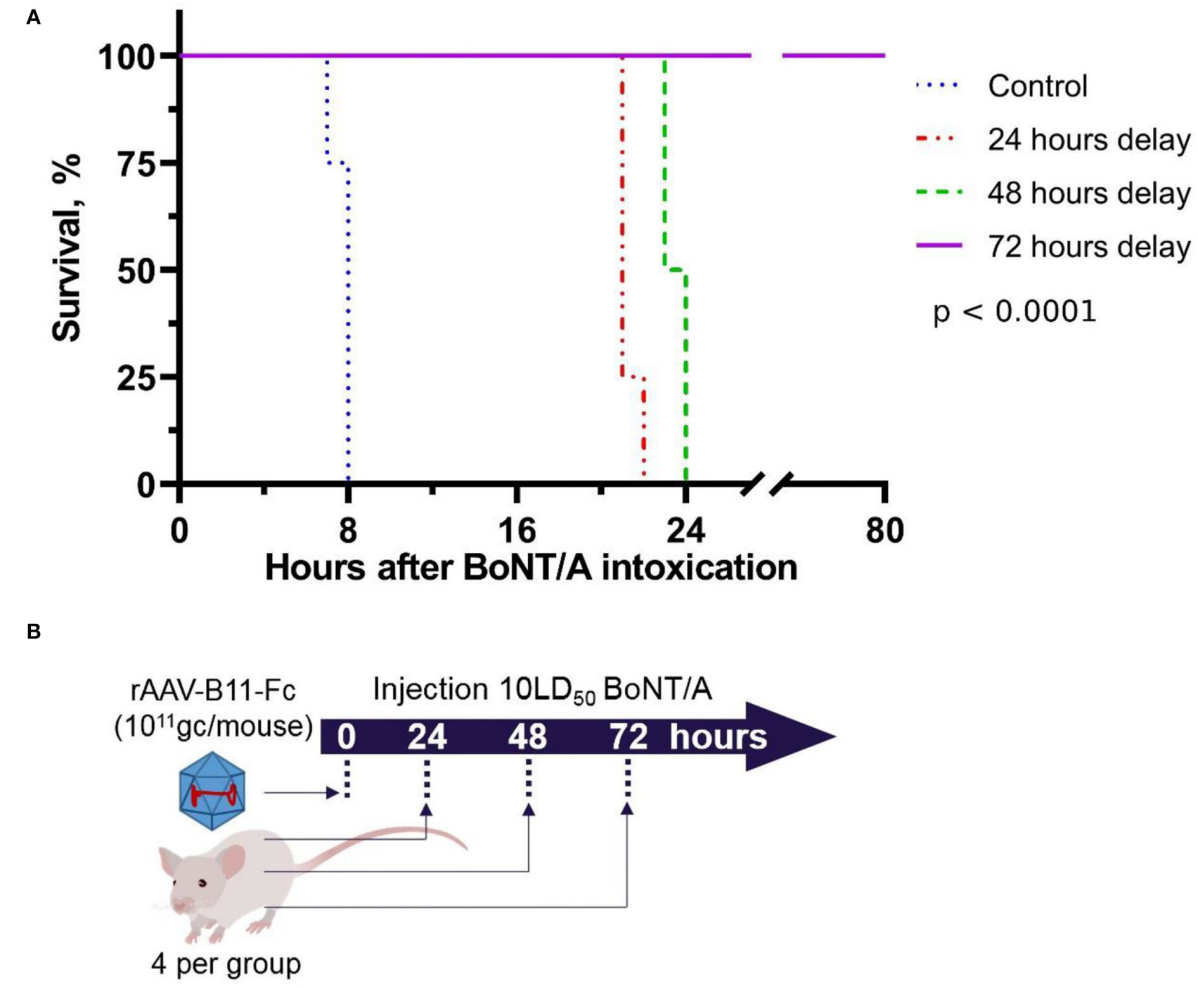
Survival of mice intoxicated with 10LD50 of BoNT/A at the 24, 48, and 72 h after intramuscular injection of rAAV-B11-Fc at a dose of 10^11^ gc/mouse. **(A)** Survival graph. Control – untreated mice, 24 h delay – mice which were intoxicated 24 h after being treated with rAAV-B11-Fc, 48 h delay – mice which were intoxicated 48 h after being treated with rAAV-B11-Fc, 72 h delay – mice which were intoxicated 72 h after being treated with rAAV-B11-Fc. The median survival time was 8 h for the control group, 21 h for the 24-h delay group, 23.5 h for the 48-h delay, and over 80 h for the 72-h delay. **(B)** Scheme of experiment.

To measure the minimum time lag between the rAAV–B11-Fc administration and toxin exposure sufficient for protection against 10LD50 of BoNT/A, mice were challenged with the toxin at the timepoints of 24, 48, and 72 h after intramuscular administration of 10^11^ gc/mouse rAAV–B11-Fc dose (four mice per point). Control mice were not injected with rAAV-B11-Fc. The scheme of experiments is shown in Figure 3B. As shown in Figure 3A, administration of rAAV–B11-Fc 72 h before BoNT/A intoxication completely protected the mice, however, mild symptoms of the disease were observed. The median survival time was longer in rAAV treated *vs* control animals and was 8, 21, 23.5, and over 80 h in the control and 24, 48, and 72 h in delay groups, respectively.

As a result of the first experiment, 3 days was estimated to be the minimal protective time lag between rAAV–B11-Fc administration at a dose of 10^11^ gc per mouse (5^*^10^12^ gc/kg) and BoNT/A intoxication was estimated to be 3 days.

**Figure 4:**
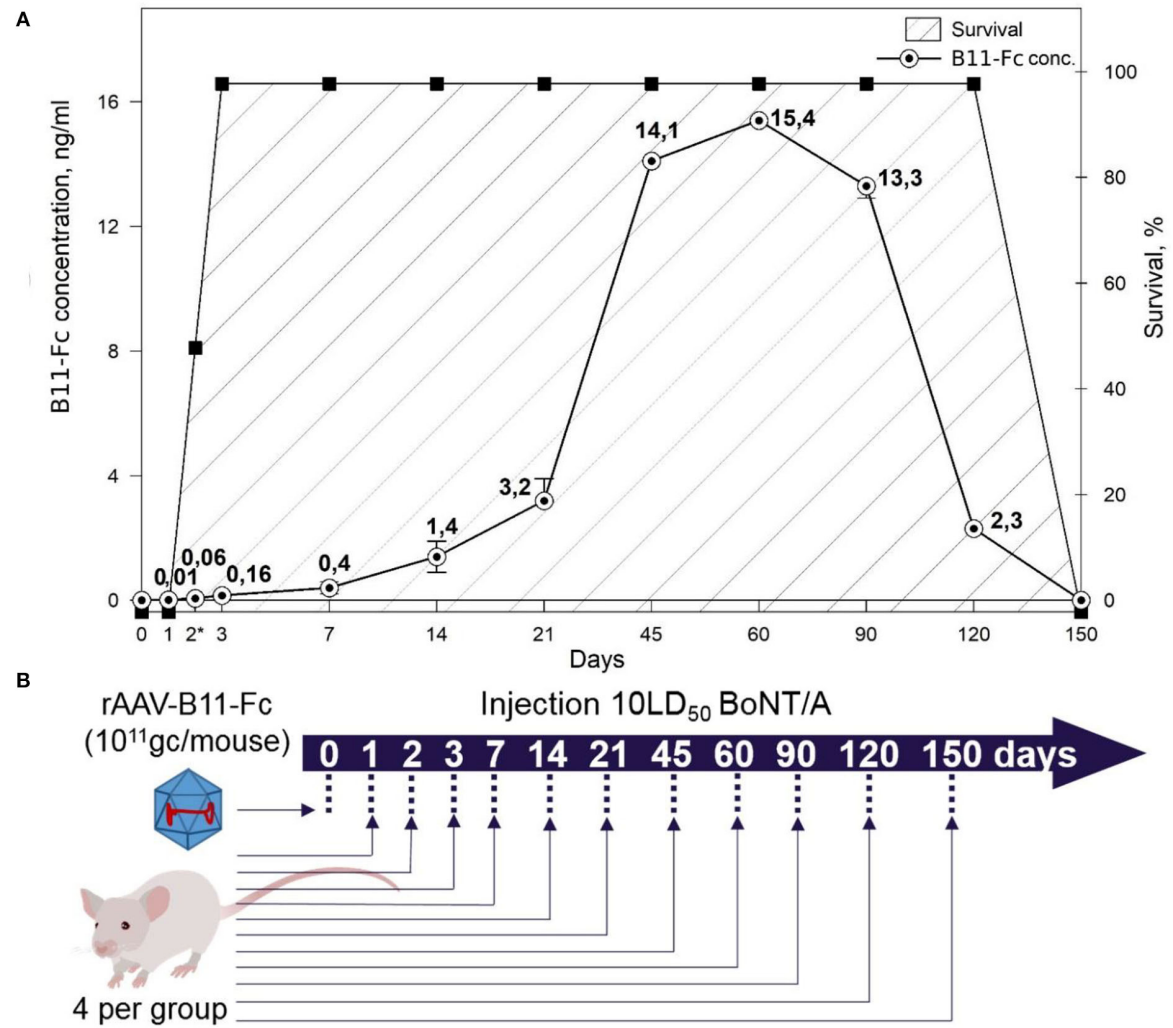
**(A)** Correlation between the concentration of B11-Fc antibodies in the serum of treated mice at various time intervals after injection of the rAAV-B11-Fc at a dose of 10^11^ gc/animal and survival of mice from 10LD50 BoNT/A. Symptoms of botulinum intoxication were monitored and marked with asterisks for partially surviving groups with mild abdominal symptoms. Survival – survival rate of mice. **(B)** Scheme of experiment.

Next, to determine the duration of protective properties of rAAV–B11-Fc against BoNT/A intoxication, many animal groups (four mice per group) were treated with rAAV–B11-Fc at a dose of 10^11^ gc, followed by intoxication with 10 LD50 BoNT/A at different time intervals. The scheme of experiments is shown in Figure 4B. The lifespan of untreated mice (control groups) after intoxication was no more than 1 day. As shown in Figure 4A, stable protection was observed starting from 3 days after rAAV–B11-Fc administration and lasted up to 120 days after treatment. There was no protective effect in the group challenged with BoNT/A on day 1 after rAAV–B11-Fc treatment, however, a 10-h delay in death was observed. In the group challenged with BoNT/A on day 2 after rAAV–B11-Fc administration, one mouse had moderate symptoms of intoxication in the form of abdominal breathing, one mouse died after 2 days and the other two mice died on the next day after the challenge. No symptoms of intoxication were observed in groups challenged on the remaining days excluding the group, challenged at day 150 (mice in this group died on the next day after the challenge).

Thus, the results of these two experiments showed that the rAAV–B11-Fc dose of 10^11^ gc per mouse (5^*^10^12^ gc/kg) can provide a significant level of protection against a lethal dose of BoNT/A, starting on day 3 after injection and lasting for at least 120 days.

### rAAV–B11-Fc exposure associated with *in vivo* expression of B11-Fc antibody

Next, the correlation between protection against BoNT/A and B11-Fc antibody concentration was determined. For this, serum samples from days 0, 1, 2, 3, 7, 14, 21, 45, 60, 90, 120, and 150 after mice treatment with rAAV–B11-Fc were evaluated for the quantity of expression of B11-Fc antibody. As shown in Figure 4A, a single intramuscular injection of rAAV–B11-Fc at a dose of 10^11^ gc/animal resulted in a concentration of 0.01, 0.06, 0.16, 0.4, 1.4, and 3.2 ng/ml of protective B11-Fc antibody on days 1, 2, 3, 7, 14, and 21, respectively.

There was a sharp increase in the expression and concentration of antibodies in the blood, up to 14.1 ng/ml between 21 and 45 days after the treatment. The maximum concentration of B11-Fc antibody was observed on day 60 and was equal to 15.4 ng/ml (~0.193 pmol/ml), after which there was a gradual decrease in concentration on day 90, and a sharp decrease on day 120. On day 150 after rAAV–B11-Fc administration, the presence of the B11-Fc antibody in the serum samples was not detected.

Thereby, the data of the pharmacokinetic experiment correlate with the data shown in Section “Study of rAAV-B11-Fc protective capacities in the prevention mode”. Thus, significant protection in mice treated with rAAV–B11-Fc begins when the B11-Fc antibody concentration reaches 0.16 ng/ml (~0.002 pmol/ml) on day 3 and continues until B11- Fc falls below 2.3 ng/ml after 120 days (~0.029 pmol/ml). Therefore, it can be assumed that the threshold protective concentration of antibodies in serum is above 0.1 ng/ml (~0.0013 pmol/ml).

### Study of rAAV–B11-Fc immunogenicity after single injection

**Figure 5:**
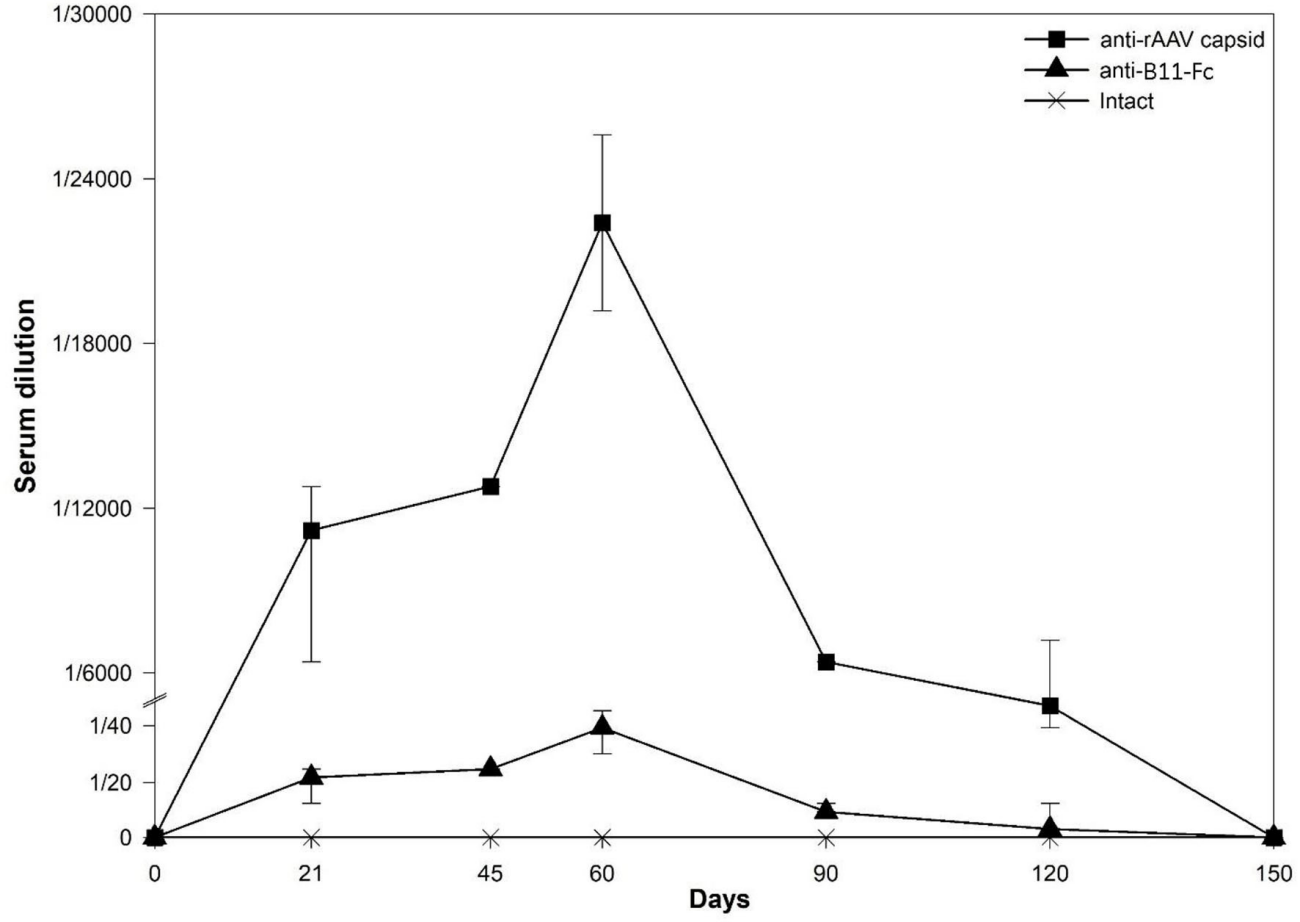
Determination of antibody concentration to expressed injected rAAV-B11-Fc antibodies (anti-B11-Fc) and antibodies to rAAV capsid proteins (anti-rAAV) following the administration of 10^11^ gc/mouse rAVV dose. Anti-rAAV capsid - the amount of anti-rAAV antibodies in mice serum, anti-B11-Fc - the amount of anti-B11-Fc antibodies in mice serum, intact - the amount of anti-B11-Fc and anti-rAAV antibodies in serum of untreated mice.

To investigate the immunogenicity of the rAAV–B11-Fc as well as the B11-Fc antibody expressed in its composition, serum samples from mice transduced with rAAV–B11-Fc were collected at various time intervals (0, 21, 45, 60, 90, 120, 150 days) and then analyzed for the presence of anti-rAAV capsid protein antibodies and anti-B11-Fc antibodies. As shown in Figure 5, a significant titer of anti-rAAV–B11-Fc antibodies was detected, and an insignificant titer of antibodies to B11-Fc, expressed *via* rAAV–B11-Fc transduction, was also detected.

The highest levels of anti-rAAV-B11-Fc and anti-B11-Fc antibodies titers were observed on day 60 and were determined as 1:12,800 and 1:50, respectively. After day 90, levels of anti-rAAV–B11-Fc and anti-B11-Fc antibodies decreased significantly and were below the limit of detection on day 150.

Thus, as a result of the experiment, rAAV–B11-Fc transduction was shown to induce humoral immune response predominantly to rAAV–B11-Fc capsid proteins and, to a small extent, to B11-Fc antibody, expressed *via* transduction.

### Determining the breakthrough dose of exposure of BoNT/A for the rAAV–B11-Fc-treated mice

**Figure 6:**
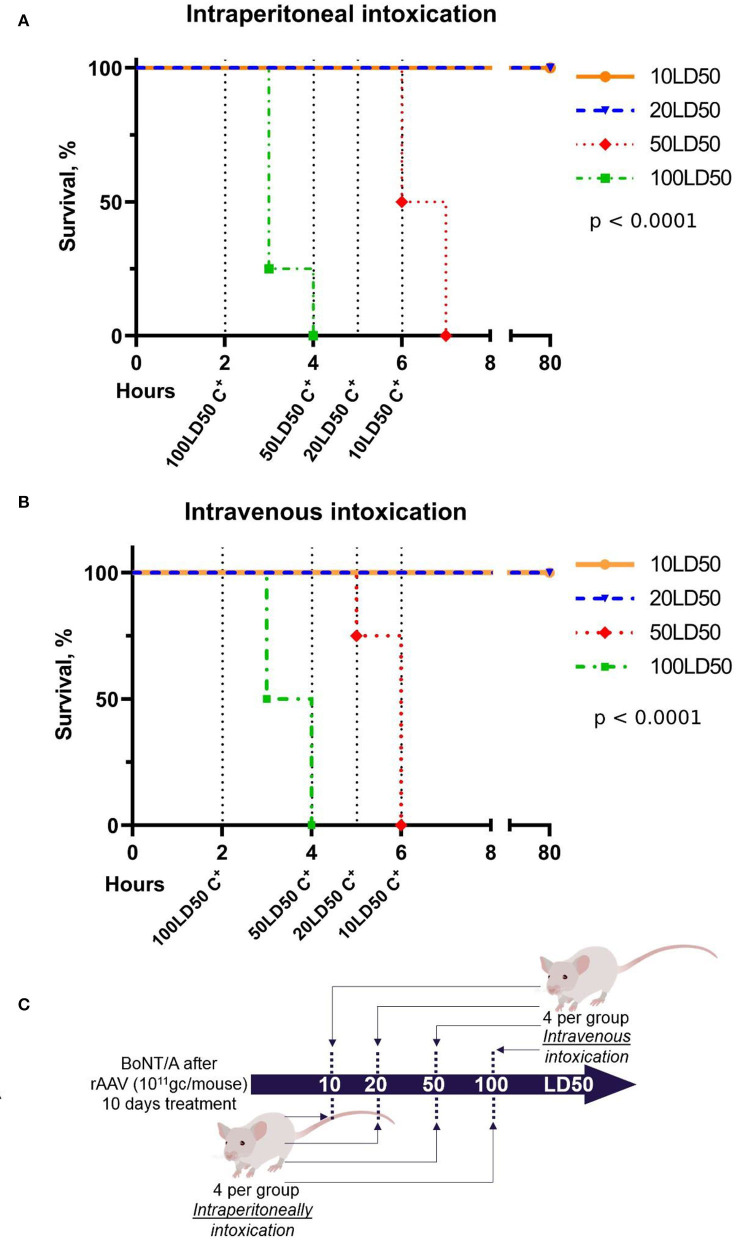
Determination of the breakthrough dose of BoNT/A in intraperitoneal **(A)** and intravenous **(B)** intoxication 10 days after the administration of rAAV-B11-Fc according to the experimental scheme **(C)**. “C^+^” on the graphs indicates the positive control of intoxication.

To determine the breakthrough dose of exposure of BoNT/A, rAAV–B11-Fc treated mice (10^11^ gc per mouse) were challenged with 10, 20, 50, and 100 LD50 BoNT/A, 10 days after rAAV–B11-Fc administration. The scheme of the experiment is shown in Figure 6C. As a result (shown in Figures 6A,B), protection against 10 and 20 LD50 was reached following both intravenous and intraperitoneal administration of BoNT/A, however, there was no protection against 50 and 100 LD50 BoNT/A. Thus, serum B11-Fc antibody level reached on day 10 following rAAV–B11-Fc administration was shown to be sufficient to protect the animals from 20 LD50 but not from 50 and 100 LD50 BoNT/A.

## Discussion

The use of neutralizing antibodies for the treatment of a variety of diseases is fairly common. For example, “Bezlotoxumab” is based on monoclonal antibodies against *Clostridium difficile* toxin B (Muyldermans, 2013; Navalkele and Chopra, 2018). However, antibody-based therapeutics are used mainly for treatment, but not for the prevention of infectious diseases, due to limited time of circulation in the body (Lu et al., 2020). Such disadvantage can be corrected by using recombinant viral vectors, which provide long-lasting expression of antibodies in the organism *via* transduction of target cells. Such a way can be used to increase antibody half-life along with Fc-fragment modifications and may be especially relevant for developing alternative means of preventing infectious diseases. There are also methods of non-viral delivery of antibody genetic materials, the results of which are described by Mukherjee et al. (2022) against three serotypes of BoNT/A from 100 LD50 toxin, but the duration of such protection was 4 days. Thus, the approach of delivering antibody genes using rAAV can be applied to increase the half-life of the B11-Fc antibody (single domain antibody fused with human IgG Fc-fragment) obtained by us in the previous study (Godakova et al., 2019) and enables induction of long-term protection. The B11-Fc antibody is a single-domain antibody fused with human IgG Fc-fragment with strong neutralizing activity against BoNT/A (Godakova et al., 2019).

Single-domain antibodies (sdAb) are the variable heavy chain fragments (VHH) of antibodies found in the Camelidae family and retain full antigenic specificity. The CDR3 region of these antibodies can form elongated structures that allow antibody interaction with hidden regions of the antigen. In addition, preparations based on VHH have higher stability, which affects the ease of production and storage (Hassanzadeh-Ghassabeh et al., 2013). VHH with modification is much easier to manipulate to increase efficiency (Mukherjee et al., 2012; Tremblay et al., 2013). It was shown that the fusion of such antibodies with the Fc fragment of the human IgG reduces the protective concentration of antibodies against 5 LD50 botulinum toxin A 100,000 times (up to 1 ng/mouse), as well as increases the half-life >100 times (up to 336 h) in the body due to an increase in the size of antibodies and a decrease in the renal filtration (Harmsen et al., 2005).

As mentioned above, the use of therapeutics based on protective antibodies (including single-domain ones) for prophylaxis requires their long-term circulation in the body. Methods for genetic delivery based on viral vectors, such as recombinant adenoviruses (Ad) (Skaricic et al., 2008; Sofer-Podesta et al., 2009) and rAAV, are used nowadays (Fang et al., 2007; Zuber et al., 2008). Previous studies were carried out on the Ad and rAAV vectors to provide prolonged preventive protection against the anthrax toxin by expressing monoclonal antibodies in their composition (Kasuya et al., 2005; De et al., 2008). These studies showed protection for 8 weeks following injection with Ad vectors, and 6 months in the composition with rAAV, which demonstrates low immunogenicity and high efficacy of rAAV. In addition, viral vectors are capable of stimulating antibody expression for passive immunotherapy (De et al., 2008; Zuber et al., 2008). Protection against botulinum toxin with VHH in the adenoviral vector has been described (Mukherjee et al., 2014), but no studies reporting the usage of rAAV vectors for the delivery of VHH have been found.

In our study, we focused on the use of the non-replicating rAAV hybrid serotype DJ, which has demonstrated therapeutic value in combining the qualities of several natural serotypes (Grimm et al., 2008), for B11-Fc antibody gene delivery. The use of rAAV as a delivery vehicle was justified by its inability to integrate into the genome, as well as long-term persistence due to its low immunogenicity (Mingozzi and High, 2013). The mechanism of rAAV long-term persistence is not fully understood, but it is assumed that rAAV does not effectively activate antigen-presenting cells (Mays et al., 2014). The obtained *de novo* rAAV–B11-Fc (Figure 1A) can transduce HEK293 cells and produce the B11-Fc antibody (Figure 1B), which has protective properties against BoNT/A *in vivo*. We demonstrated that a single intramuscular injection of rAAV–B11-Fc acts rapidly to protect mice from subsequent BoNT/A intoxication with an effect lasting for several months and, confirming the efficacy of rAAV–B11-Fc against BoNT/A. The intramuscular way of rAAV–B11-Fc administration was chosen because skeletal muscle cells are non-dividing cells (Alberts et al., 2002), which allows long-term persistence of rAAV–B11-Fc without loss of expression. The choice of different rAAV–B11-Fc experimental doses was based on the data obtained by other researchers earlier when rAAV was used for the treatment of various diseases (Pien et al., 2009; Hsu and Safdar, 2011; Balazs et al., 2013; Greig et al., 2014; Hanlon et al., 2019). A dose of 10^11^ gc/mouse protected mice as early as day 3 (Figure 2), while lower doses caused later protection, which could be due to insufficient accumulation of the expressed antibodies. Administration of a 10^11^ gc/mouse rAAV–B11-Fc dose 24 and 48 h before intoxication showed a significant survival time increase in BoNT/A-intoxicated mice (Figure 3A).

Complete protection of rAAV–B11-Fc-transduced mice (10^11^ gc per mouse) was observed during BoNT/A intoxication 72 h after transduction. The protective effect of B11-Fc antibody against 10 LD50 (~0.855 pmol/ml) toxin was achieved on day 3, when its concentration in mouse serum reached 0.16 ng/ml (~0.002 pmol/ml; Figure 4A). Thus, 0.002 pmol of antibodies was shown to fully detoxify 0.855 pmol of the toxin, which indicates a high neutralizing potency of the B11-Fc antibody expressed by rAAV–B11-Fc against BoNT/A intoxication. These results are consistent with the neutralizing activity of similar antibodies (Venkatasubramaniam et al., 2019). Since the peak of B11-Fc antibody concentration in the serum samples of rAAV–B11-Fc-transduced mice was 15.4 ng/ml (~0.193 pmol/ml) at day 60 (Figure 4A), it can be assumed that at this time point the animals might be protected from a higher dose of BoNT/A intoxication. The protective effect of rAAV–B11-Fc at a dose of 10^11^ gc/mouse was observed up to 120 days after the administration, which indicates the capability of the B11-Fc antibody expressed in the rAAV vector to provide much longer protection against BoNT/A intoxication compared to purified B11-Fc antibody administered parenterally.

While investigating the breakthrough dose of botulinum toxin on day 10 after rAAV–B11-Fc injection, when the concentration of B11-Fc antibody in the serum was ~1 ng/ml, mice were shown to be protected from 20 LD50 of BoNT/A, but not from 50 and 100 LD50 BoNT/A. Thus, the data show the inability of rAAV–B11-Fc to protect against high doses (50 and 100 LD50) of BoNT/A within the first 2 weeks after administration. However, taking into account the results of the B11-Fc antibody concentration at various time intervals after rAAV–B11-Fc administration, as well as the dependence of protection against BoNT/A on the concentration of B11-Fc antibody in the serum, it can be assumed that the effective protection of animals against breakthrough doses (50 and 100 LD50) of BoNT/A will start ~21 days and will end between 90 and 120 days after injection.

Although there is evidence that VHH is non-immunogenic (Hassanzadeh-Ghassabeh et al., 2013; Muyldermans, 2013), the immunogenicity of a recombinant VHH-Fc antibody expressed *via* rAAV transduction had to be investigated. Our results demonstrate that anti-B11-Fc antibodies can be detected in the serum samples of rAAV–B11-Fc-transduced mice in a small quantity, with a peak concentration of 1/50 on day 60 (Figure 5). This observation may be related to the presence of a human IgG Fc-fragment, which might be immunogenic for mice. However, despite this, anti-B11-Fc antibody titers are low and do not seem to affect the duration and level of protection of rAAV–B11-Fc-transduced mice. In addition, anti-B11-Fc antibodies can theoretically enhance the elimination of the toxin due to the binding of the B11-Fc+BoNT/A immune complex and its elimination due to Fc-mediated functions.

When studying the immune response to the rAAV capsid proteins in the serum of transduced mice, it was demonstrated that the level of antibodies to the rAAV vector increases sharply already on day 21 (Figure 5), significantly decreases by day 90, and drops below quantification limit on day 150 after rAAV–B11-Fc transduction. These data are consistent with previous studies where AAV8 administration of 2^*^10^12^ gc/kg increased capsid-specific antibodies reaching the peak at 7–9 weeks after administration (Nathwani et al., 2011; Mingozzi and High, 2013). The presence of antibodies to rAAV may be important and contribute to the elimination of rAAV-B11-Fc from the body, which may explain the decrease in the level of B11-Fc antibody circulation in the serum of transduced animals after 60 days, and is also observed in similar studies (Wang and Huang, 2000; Ronzitti et al., 2020).

In general, our studies suggest that antibodies expressed *via* rAAV transduction can be present in the body for a long time and provide long-term protection against a lethal dose of BoNT/A. A single intramuscular injection of rAAV–B11-Fc showed protection of mice against botulinum toxin (10 LD50) starting on day 3 and lasting for several months. Thus, this approach can serve as the basis for the development of specific anti-botulinum prophylaxis agents.

An AAV formulation, therefore, has an advantage over monoclonal antibodies, characterized by a limited half-life ranging from 10 to 20 days (Ovacik and Lin, 2018), and existing vaccines against botulism with unproven clinical efficacy. As for the B11-Fc antibody, in our previous work (Godakova et al., 2019) we showed its complete elimination from the serum within 14 days after injection, while the presence of the B11-Fc antibody in the serum of rAAV–B11-Fc-transduced mice present for at least 4 months.

Based on the results obtained in this study, repeated AAV administration may reduce their efficacy due to the formation of anti-capsid antibodies, however, if repeated protection is necessary, there is the possibility of changing the AAV serotype (Rivière et al., 2006). The presence of antibodies to the AAV makes the reuse of preparations based on them problematic impairing the efficiency of transduction and transgene expression. Among the options for solving this problem are (Mingozzi and High, 2013): introduction of higher doses of the vector, replacement of the AAV serotype, introduction of empty capsids for anti-AAV antibodies adsorption, or the use of plasmapheresis. Each option has positive aspects and they can be combined to achieve the best result. The use of pegylation or encapsulation of AAV in polymer gels enables vector protection from neutralization (Sailaja et al., 2002; Lee et al., 2005) and is currently considered to be one of the most prospective strategies to maintain AAV activity. At the same time, an immune response against B11-Fc can be favorable because it has been shown to accelerate the elimination of the antibody-bound toxin and therefore can reduce toxin concentration in the body (Sepulveda et al., 2010).

Thus, the results of our studies show that rAAV can be effectively used for the delivery and long-term expression of neutralizing antibodies *in vivo*, providing long-term protection against intoxication caused by botulinum neurotoxin type A.

## Data availability statement

The original contributions presented in the study are included in the article/supplementary material, further inquiries can be directed to the corresponding author/s.

## Ethics statement

The animal study was reviewed and approved by Institutional Animal Care and Use Committee (IACUC) of the Federal Research Centre of Epidemiology and Microbiology Named after Honorary Academician N. F. Gamaleya.

## Author contributions

Conceptualization: IE, DS, AN, and BN. Methodology: ER, IE, AD, AN, DL, and IV. Validation and formal analysis: ER, IE, and AD. Investigation: ER, AD, and SG. Resources: DS, BN, and AG. Project administration: DS, BN, DL, and AG. Data curation and supervision: IE. Writing—original draft preparation: AD and ER. Writing—review and editing: IE, DS, and AN. Visualization: AD and IE. All authors contributed to the article and approved the submitted version.

## Conflict of interest

The authors declare that the research was conducted in the absence of any commercial or financial relationships that could be construed as a potential conflict of interest.

## Publisher's note

All claims expressed in this article are solely those of the authors and do not necessarily represent those of their affiliated organizations, or those of the publisher, the editors and the reviewers. Any product that may be evaluated in this article, or claim that may be made by its manufacturer, is not guaranteed or endorsed by the publisher.
